# Vertical distribution characteristics and source apportionment of nitrogen in the Longyangxia Reservoir in the upper reaches of the Yellow River

**DOI:** 10.1371/journal.pone.0326038

**Published:** 2025-06-16

**Authors:** Wei Wu, Yuhe Dong, Chen Li, Hang Chen, Lei Ren, Sheng Xu

**Affiliations:** State Key Laboratory of Eco-hydraulics in Northwest Arid Region of China, Xi’an University of Technology, Xi’an, Shaanxi, China; Universidade Catolica Portuguesa, PORTUGAL

## Abstract

Studying the biogeochemical cycle of biogenic nitrogen and its influence on hydrological processes and anthropogenic nitrogen input is of great significance for water resource management and the maintenance of aquatic ecosystems in ecologically sensitive areas. Currently, there is a limited understanding of the sources contributing to nitrate levels during thermal stratification in deep and large reservoirs, as well as the transformation processes of nitrate under varying hydrological conditions. This study collected water samples from the Longyangxia Reservoir, located in the upper reaches of the Yellow River, during January and April of 2024. Utilizing hydrogeochemical analysis, multivariate stable isotope technology, the Bayesian isotope mixing model, and multivariate statistical analysis, we analyzed the vertical distribution characteristics of nitrogen in the reservoir across different periods. The transformations and sources of nitrogen were identified, and the contribution rates of each nitrogen source were estimated. The results indicate that January serves as the mixing period for the Longyangxia Reservoir, during which the differences in nitrogen concentration among the vertical water layers are relatively minimal. The concentration ranges for nitrate (NO₃⁻), dissolved organic nitrogen (DON), and ammonium (NH₄⁺) were observed to be 0.598–0.647 mg/L, 0.124–0.397 mg/L, and 0.015–0.157 mg/L, respectively. Beginning in April, the reservoir enters the thermal stratification period, characterized by higher concentrations of various nitrogen forms compared to the mixing period. During the stratification period, the concentration of various nitrogen forms within the vertical profile of the reservoir demonstrates a characteristic distribution of being low in the upper section, maximum values of total nitrogen (TN) and dissolved DON in the middle section, and maximum concentrations of NO₃⁻ and NH₄⁺ in the bottom section. Nitrate nitrogen and dissolved organic nitrogen are the primary forms of nitrogen present in the Longyangxia Reservoir, constituting 66.71% and 25.83% of the total dissolved nitrogen in January, and 62.39% and 21.59% in April, respectively. During the sampling period at Longyangxia Reservoir, the δ^15^N-NO_3_^-^ values in the water ranged from 5.58 ‰ to 7.38 ‰, while the δ^18^O-NO_3_^-^ values varied from −5.87 ‰ to 2.58 ‰. Nitrification is identified as the primary nitrogen conversion process occurring in the reservoir water. Under aerobic conditions, denitrification does not occur in aquatic environments. The dynamics of nitrate in the bottom layer are influenced by nitrification processes and the release of nitrogen from sediment. Soil organic nitrogen is the primary source of nitrate in Longyangxia water, contributing 42.1% and 51.8% during the sampling period, respectively. This study introduced sediment as an additional end member, highlighting that the contribution of sediment to nitrate in water is significant, accounting for 24% and 14.1%, respectively. This study offers valuable insights for precise nitrogen management and control in deep reservoirs by tracking nitrate sources and quantifying their contributions.

## Introduction

In the past few decades, approximately 58,000 large dams have been constructed worldwide [1]. These large dams play an increasingly significant role in hydroelectric power generation, flood control, irrigation, and the supply of drinking water [2,3]. The construction of dams disrupts the connectivity of rivers, resulting in reduced flow velocity, extended water retention time, and alterations in redox conditions [4]. These changes directly impact the biogeochemical cycles of river ecosystems [5,6]. Nitrate is a crucial biogenic substance in ecological processes, playing a significant role in the biochemical cycling within aquatic systems [7,8]. However, excessive input of nitrate not only results in the rapid proliferation of algae and other phytoplankton, leading to the eutrophication of water bodies, but also generates the potent greenhouse gas Nitrous Oxide (N_2_O) during its transformation, which poses a serious threat to the regional ecological environment [9,10]. Understanding the distribution of nutrients, as well as the sources and transformations of nitrate in large reservoirs, is essential for the sustainable utilization of water resources and the maintenance of ecosystems.

Deep reservoirs are susceptible to developing a thermal stratification structure during warm seasons, which comprises the epilimnion, metalimnion, and hypolimnion [11,12]. Thermal stratification can significantly influence the migration and transformation processes of nitrate [13]. Wang discovered that the Hongfeng Reservoir, situated in southwestern China, exhibits a pronounced stratification phenomenon during the flood season. This stratification is influenced by various factors, including hydrodynamic conditions and dissolved oxygen levels. Nitrification was observed in the water column up to a depth of 10 meters, whereas denitrification occurred at the lake’s bottom [14]. In the Changzhao Reservoir located in Zhejiang Province, aerobic conditions prevailing in a low phosphorus environment inhibit denitrification [15]. Generally, the dynamics of nitrate in lakes are influenced by factors such as climatic conditions, lake morphology, biogeochemical processes, and human activities [16,17].

Nitrates derived from various sources exhibit distinct nitrogen and oxygen isotope characteristics. A series of biochemical reactions, including mineralization, assimilation, nitrification, and denitrification, that occur within the natural nitrogen cycle, correspondingly result in alterations in the stable nitrogen and oxygen isotope ratios [18]. Furthermore, the contribution ratios of different potential nitrate sources can be quantified utilizing Bayesian isotope mixing models. The dual stable isotope method for nitrate (δ15N-NO_3_-, δ18O-NO_3_-) has been effectively applied in numerous watersheds as a reliable approach for tracing the dynamics and sources of nitrate [19–23]. However, most current research on the kinetics and sources of nitrate is conducted in shallow water or groundwater, with limited studies focusing on nitrogen and oxygen isotopes in deep reservoirs.

This study investigates the nutrient concentrations and nitrogen and oxygen isotope values in the Longyangxia Reservoir (LYXR), the largest reservoir in the Yellow River Basin, during January and April 2024. LYXR is a deep reservoir. Investigating the distribution characteristics and sources of nitrate in its water body is significant for maintaining the ecosystem of the basin. The primary objective of this study is to clarify the distribution characteristics of the nitrogen-related physical and chemical indicators in the Longyangxia Reservoir, understand the impact of thermal stratification on nitrate dynamics, explore the sources and transformation processes of nitrate in reservoirs, and quantitatively evaluate the contribution rate of nitrate from various sources to water bodies. This research aims to enhance the scientific and effective management of the reservoir water environment and provide a scientific basis for protecting the ecological environment of the upstream Yellow River.

## Materials and methods

### Study area

The Longyangxia Reservoir is situated in the Longyangxia Gorge, straddling the border between Gonghe County and Guinan County in Qinghai Province. It is the nearest large reservoir with multi-year regulation capacity to the source area of the Yellow River, managing 65% of the water volume in the upper reaches of the river. The reservoir begins storing water in May and releases water from November to May of the following year, establishing an operational mode characterized by ‘abundant storage and dry discharge’ [24]. The designed water level is set at 2600 meters, with a total storage capacity of 24.7 billion cubic meters and a regulating storage capacity of 19.4 billion cubic meters. This infrastructure provides essential drinking water and hydropower for the local residents. The average depth of the reservoir is 65 meters, with a maximum depth of 137 meters. Following the construction of the Longyangxia Reservoir, the average annual inflow is recorded at 622.81 m³/s, while the average annual outflow is 538.06 m³/s. The water intake system of the reservoir comprises several layers of intake channels, including bottom holes, deep holes, and middle holes. The discharge from the middle hole is a widely employed method for water intake. The average altitude of the research area is 2,700 meters, making it the highest artificial lake in China. This area is characterized by a plateau continental climate, with an average annual temperature of 7.5 °C and a maximum water temperature of 16.5 °C in the lake. Rainfall is predominantly concentrated from June to August, with an average annual precipitation ranging from 250 to 450 mm, while the average annual evaporation is between 1,101.7 and 1,322.5 mm. The research area is situated in a county that primarily emphasizes animal husbandry, which is integrated with agricultural practices. The predominant livestock raised includes yaks and Tibetan sheep. The region features natural grasslands, river valleys, and small agricultural fields, where crops such as barley and corn are mainly cultivated. The research area is characterized by unique geographical conditions, significant environmental heterogeneity and sensitivity, and is highly susceptible to both natural factors and human activities.

### Sample collection and analysis

**Fig 1:**
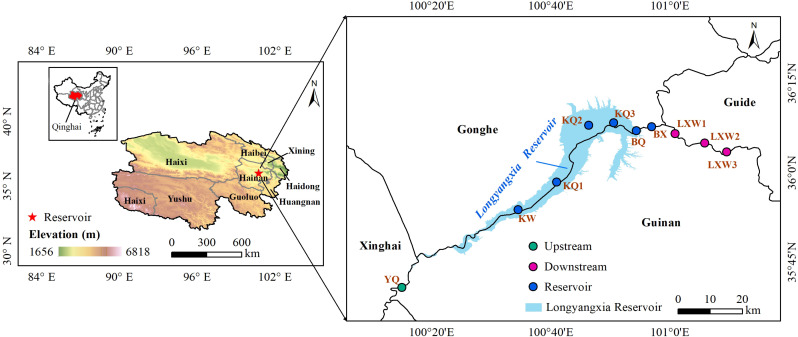
Distribution of sampling points in the Longyangxia Reservoir. The map used in this study was generated using ArcGIS 10.8. The elevation data for the study area, SRTM 90m DEM, was obtained from the Earth Resource Data Cloud website (www.gis5g.com). During the sampling process, we strictly followed the standardized operating procedures (Water quality—Guidance on sampling techniques (HJ 494-2009), issued by the Ministry of Environmental Protection of the People’s Republic of China). Parallel sampling was conducted at all sampling sites to ensure the consistency and representativeness of the samples. The collected data were subject to preliminary review, and any anomalies were rechecked and resampled.

This study conducted surface water sampling in January and April 2024 at both the upstream (YQ) and downstream (LXW1-LXW3) locations of LYXR, as well as at various points including below the dam (BX), in front of the dam (BQ), within the reservoir area (KQ1-KQ3), and at the reservoir tail (KW). Vertical water samples were collected in front of the dam at depths of 5, 10, 20, 40, 60, 80, and 100 meters, with the vertical sampling site located approximately 50 meters from the dam (Fig 1). Surface water samples were collected at a depth of 0.5 meters below the water surface using an organic glass water sampler. Additionally, layered water samples were obtained using a Niskin layered water sampler from the Danish company KC Denmark, following a predetermined vertical line in front of the dam. Additionally, potential sources of nitrate in the water of Longyangxia Reservoir, including soil and sediment, were collected. Two soil samples were collected from the surrounding study area at a depth of 0−10 cm using a handheld shovel. Two sediment samples from the reservoir were collected using a column sampler and a mud grab bucket. After freezing and air drying, the solid samples were ground and sieved through a 100-mesh sieve. Subsequently, 0.3 g of the ground and sieved solid sample was weighed, and 3 mL of ultrapure water was added. The sample was then placed in an ultrasonic cleaning machine (Xinzhi SB25−12DDT) for ultrasonic extraction for 10 minutes to obtain the supernatant. The supernatant was filtered and transferred to a test tube for further analysis.

The water temperature (WT), dissolved oxygen (DO), and acidity (pH) were measured on-site using a portable multiparameter water quality analyzer (Hach HQ40d). Subsequently, the untreated water sample was stored in 500 mL polyethylene bottles, placed in a −20 °C refrigerator, and promptly transported back to the laboratory for processing. The water sample is filtered through a 0.45 μm filter membrane to determine chlorophyll a (Chl-a), while the filtered water sample is utilized for the determination of total dissolved nitrogen (TDN). Dissolved organic nitrogen (DON) is calculated by subtracting dissolved inorganic nitrogen from TDN [25]. The concentrations of nitrate (NO_3_-), ammonium (NH_4_+) and nitrite (NO_2_-) were measured using a portable multiparameter water quality analyzer (LH-C600, Lianhua Technology), which has minimum detection limits of 0.1 mg/L for NO_3_-, 0.01 mg/L for NH_4_+, and 0.003 mg/L for NO_2_-, with a testing accuracy of ± 8%.

The water samples utilized for isotope determination are filtered through a 0.45 μm membrane. Isotope indices were determined using a stable isotope ratio mass spectrometer (MAT253, Thermo Fisher, USA). Specifically, nitrogen and oxygen isotopes (δ15N-NO_3_-, δ18O-NO_3_-) were analyzed utilizing the bacterial denitrification method [26]. In this process, specific denitrifying bacteria are employed to concentrate and package the bacterial solution after it has passed a colorimetric test. Subsequently, the solution is purged with high-purity nitrogen (N_2_). The thawed standard solution and sample are added to the bacterial solution using an airtight syringe for an overnight reaction, facilitating the quantitative conversion of NO_3_- to N_2_O. The following day, sodium hydroxide (NaOH) is added to terminate the reaction and absorb carbon dioxide (CO_2_), after which the generated N_2_O is analyzed using the mass spectrometer. The analysis accuracies of δ15N-NO_3_-, δ18O-NO_3_- values are ± 0.5 ‰ and 1 ‰, respectively. The oxygen isotope values (δ18O-H_2_O) of the water sample were determined using a high-temperature pyrolysis stable isotope mass spectrometer (Flash EA 1112 HT Dela V Advantages, Thermo Fisher, USA). The accuracy of the δ18O-H_2_O analysis is ± 0.3 ‰. The relative ratio of isotopes is represented by δ:


$$\delta (‰)=\left[\frac{R_{\text{sample}}-R_{\text{standard}}}{R_{\text{standard}}}\right]\times 1000$$ (1)

Where $R_{sample}$ and $R_{s\tan dard}$ are the ratios (^15^N/^14^N or ^18^O/^16^O) of the sample and standard, respectively. The nitrogen isotope is referenced to atmospheric nitrogen, with an R_standard_ (^15^N/^14^N_air_) value of 0.0036765. The oxygen isotope is referenced to the Vienna Standard Mean Ocean Water (VSMOW), with an R_standard_ (^18^O/^16^O_VSMOW_) value of 0.0020052. The positive and negative values of δ indicate the enrichment and depletion of heavy isotopes in the test sample compared to the standard sample, respectively.

### SIAR model

In this study, the Bayesian isotope mixing model, SIAR, was used to estimate the sources of nitrate in LYXR water. The model utilizes Bayesian principles to determine the probability distribution of the contribution rate of each source. It comprehensively accounts for uncertainty factors associated with multiple sources, fractionation effects, and isotope characteristics, thereby enhancing the accuracy of pollution source analysis [18]. The model expression is as follows [27]:


$$\mathrm{\backslash [}X_{ij}=\sum {\mathrm{\backslash nolimits}}_{k=1}^{k}p_{k}\left(S_{jk}+C_{jk}\right)+\varepsilon_{jk}\mathrm{\backslash ]}$$ (2)

$$\mathrm{\backslash [}S_{jk}=N\left(\mu_{jk},\omega_{jk}^{2}\right)\mathrm{\backslash ]}$$

$$\mathrm{\backslash [}C_{jk}=N\left(\lambda_{jk},\tau_{jk}^{2}\right)\mathrm{\backslash ]}$$

$$\mathrm{\backslash [}\varepsilon_{jk}=N\left(0,\sigma_{j}^{2}\right)\mathrm{\backslash ]}$$

W $X_{ij}$ denotes the j^th^ isotope value for the i^th^ $S_{jk}$ denotes the j^th^ isotope value of the k^th^$\mu_{jk}$ $\omega_{jk}$ $p_{k}$ is the contribution of the k^th^ $C_{jk}$ is the fractionation factor of isotope j on the k^th^ $C_{jk}$ $\lambda_{jk}$ $\tau_{jk}$ are the mean and standard deviation of the normal distribution, respectively; $\varepsilon_{jk}$ is the residual, which represents the unquantified variation among the mixtures, with a mean of 0 and a standard deviation of $\sigma_{j}$.

In this study, potential nitrogen sources, including soil and sediment, were collected from the LYXR. All samples were subjected to parallel sampling and detection to obtain mean values, thereby minimizing errors. Based on the research results of δ^15^N-NO_3_^-^ and δ^18^O-NO_3_^-^ from various sources (Table 1), the SIAR model calculates the contribution ratios of different sources to nitrate.

**Table 1:** Characteristics of δ^15^N-NO_3_^-^ and δ^18^O-NO_3_^-^ values from different sources.

Source	Precipitation^a^	Fertilizer^a^	Soil^*^	Sediment^*^
δ^15^N-NO_3_^-^(‰)	1.4 ± 2.4	0.3 ± 3.0	5.4 ± 1.5	2.3 ± 1.1
δ^18^O-NO_3_^-^(‰)	38.5 ± 13.4	3.0 ± 1.7	−6.8 ± 1.7	5.5 ± 2.0

* The isotope data is derived from actual measurements in this study.

^a^The isotope data is referenced from previous literature [28,29].

### Statistical analysis

In this study, ArcGIS 10.8 was utilized to conduct the mapping work related to geographic information. For statistical analysis and data visualization, the primary tools employed were IBM SPSS Statistics, Excel 2021, and Origin 2022. Correlation analysis and linear regression analysis were performed with IBM SPSS Statistics 27.0. According to Shapiro-Wilk test, nonparametric test is performed when the value does not conform to the normal distribution. The quantitative calculation of nitrate sources was conducted using the SIAR package in R Studio.

## Results

### Distribution characteristics of physicochemical indicators in the Longyangxia Reservoir

**Fig 2:**
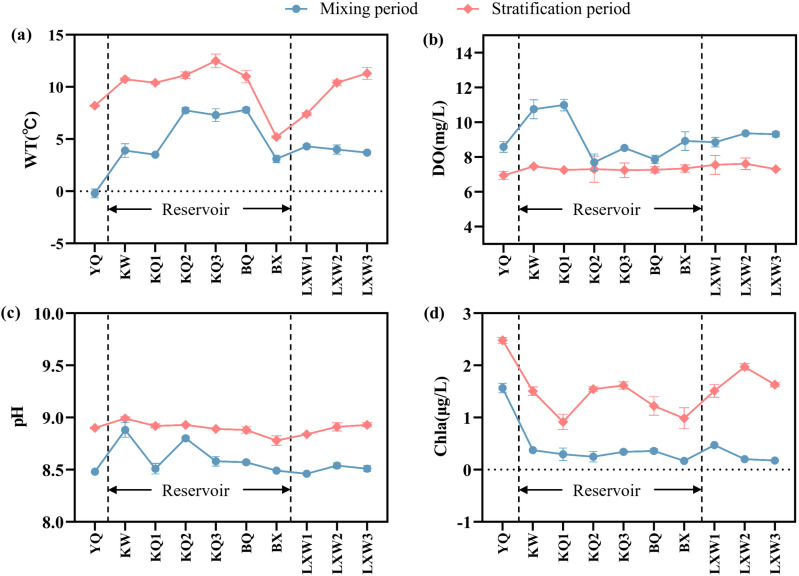
Longitudinal distribution of water temperature, dissolved oxygen, pH, and chlorophyll a in the Longyangxia Reservoir.

**Fig 3:**
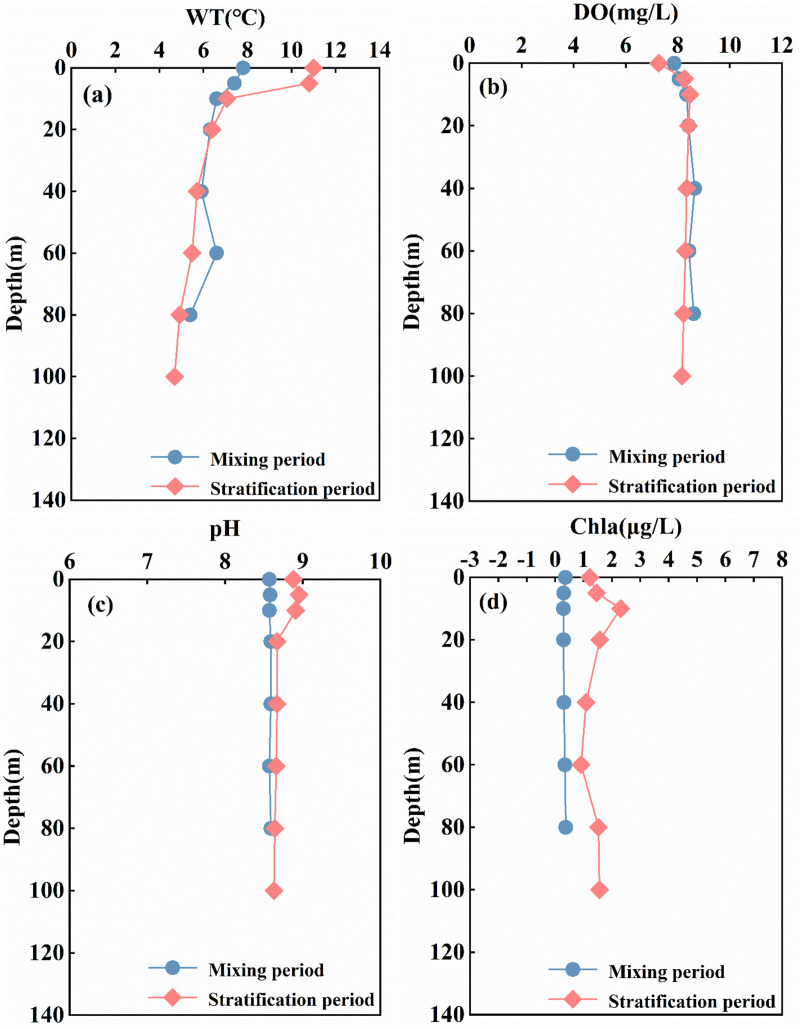
Vertical distributions of water temperature, dissolved oxygen, pH and chlorophyll a in the Longyangxia Reservoir.

The average surface water temperature of the reservoir in January was 5.56 ± 2.28 °C, rising to 10.16 ± 2.53 °C by April. The water temperature in the upstream river section is lower than that in the reservoir section, which can be attributed to factors such as terrain, altitude, and hydrodynamic conditions (Fig 2a). According to the monitoring of vertical water temperatures in front of the dam within the study area, the range of vertical water temperatures in January is between 5.4 °C and 7.8 °C, with an average temperature of 6.57 ± 0.83 °C. The vertical temperature variation is minimal. In April, the enhanced solar radiation gradually increased the surface water temperature, which stabilized at approximately 10.9 °C in the upper layer (0–5 m). In the middle layer (5–20 m), the water temperature exhibited a rapid decline from 10.8 °C to 6.4 °C at a rate of 0.3 °C/m, followed by a more gradual decrease with depth until the bottom layer (80–100 m), where it reached a stable minimum of 4.68 °C. The vertical water temperature variation of the reservoir ranges from 4.55 °C to 11 °C, with a maximum temperature difference of 6.45 °C observed between the surface and bottom water bodies (Fig 3a). The thermal stratification structure is classified based on the criterion that the water temperature in the thermocline decreases at a rate of 0.2°C/m decreases [30]. In April, weak stratification began to manifest at depths of 5–20 meters, indicating the onset of the thermal stratification period. In January, the water temperature within the reservoir exhibited a nearly isothermal distribution, indicating the mixing period.

The dissolved oxygen content is influenced by multiple factors, including hydrodynamic conditions and water temperature [31]. In January, the upstream and downstream river sections exhibit high DO levels due to increased water fluidity, whereas the reservoir section remains relatively stable due to the hydrodynamic conditions created by the dam, resulting in lower DO levels. The average DO content in the reservoir during January was 9.12 ± 1.43 mg/L, while the DO content in April stabilized at approximately 7.3 ± 0.08 mg/L (Fig 2b). In the vertical profile of the water body, the DO content exhibited a slight increase at depths of 0–20 meters, followed by a stabilization phase, and ultimately showed a decline at the bottom (Fig 3b). As an indicator of phytoplankton biomass, the Chl-a content remained relatively stable in January but exhibited a significant increase in April (Fig 2d). The concentration of Chl-a in the surface layer (0–10 m) of the reservoir increases with depth, reaching a maximum value of 2.31 µg/l near the thermocline at 10 m. Below a depth of 10 m, the concentration of Chl-a decreases with increasing water depth, stabilizing at approximately 1.56 µg/l at a depth of 80 m (Fig 3d).

According to previous studies, the phenomenon of minimal dissolved oxygen often occurs in the thermocline during the stratification period of deep and large reservoirs. This phenomenon is attributed to the oxygen consumption resulting from organic matter degradation and the obstruction of vertical exchange, which is widespread [13,31]. In this study, the DO levels in the reservoir thermocline exhibited an unexpected increase, which diverges from conventional understanding. This phenomenon may be attributed to the growth and metabolic activities of plankton and bacterial communities within the unique environment characterized by high altitude, cold temperatures, and intense solar radiation. The complex regulatory mechanisms underlying these changes warrant further investigation. In our study reservoir, we observed the phenomenon of a Deep Chlorophyll a Maximum (DCM) occurring at a depth of 10 meters. DCM is a common characteristic of stratified aquatic systems [32,33]. Light must penetrate below the surface mixing layer to form the DCM. The primary factors influencing DCM formation include the availability of light, the vertical distribution of nutrients, and the positioning of the thermal gradient within the water column [34,35]. The study reservoir was in the initial stage of thermal stratification in April, and the water stratification structure had not yet reached a completely stable state. The availability of light has a more significant effect on the vertical distribution of DO and Chl-a than the thermal gradient in the water column [32]. Previous studies indicate that phytoplankton position themselves to balance the light demand from the surface and the nutrient demand from the deeper water column. This strategy allows them to optimize the favorable conditions presented by both the upper and lower water layers. The surface layer and the thermocline exhibit unique physicochemical conditions that enable phytoplankton to ‘attend to both above and below’ [33,36]. The Chl-a content exhibited an increasing trend within the water depth range of 0–20 m, reaching a maximum concentration of 2.31 μg/L at a depth of approximately 10 m. The photosynthetic activity of a significant number of phytoplankton in the 0–20 m depth range contributed to the rise in dissolved oxygen concentration in the water. In the study area, the pH value remained between 8 and 9, indicating that the water body was weakly alkaline (Fig 2c and 3c).

### Distribution characteristics of nitrogen in the Longyangxia Reservoir

**Fig 4:**
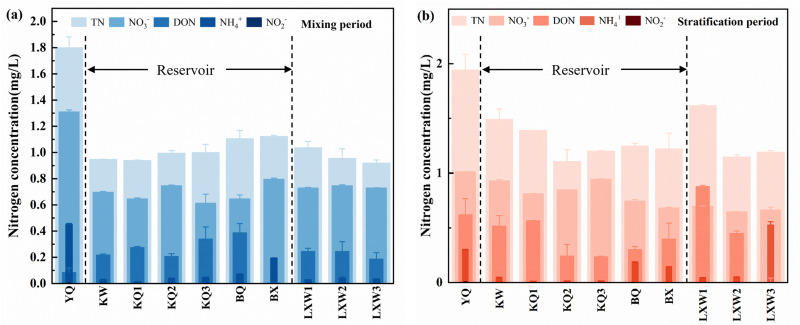
Longitudinal distribution characteristics of nitrogen in the Longyangxia Reservoir.

**Fig 5:**
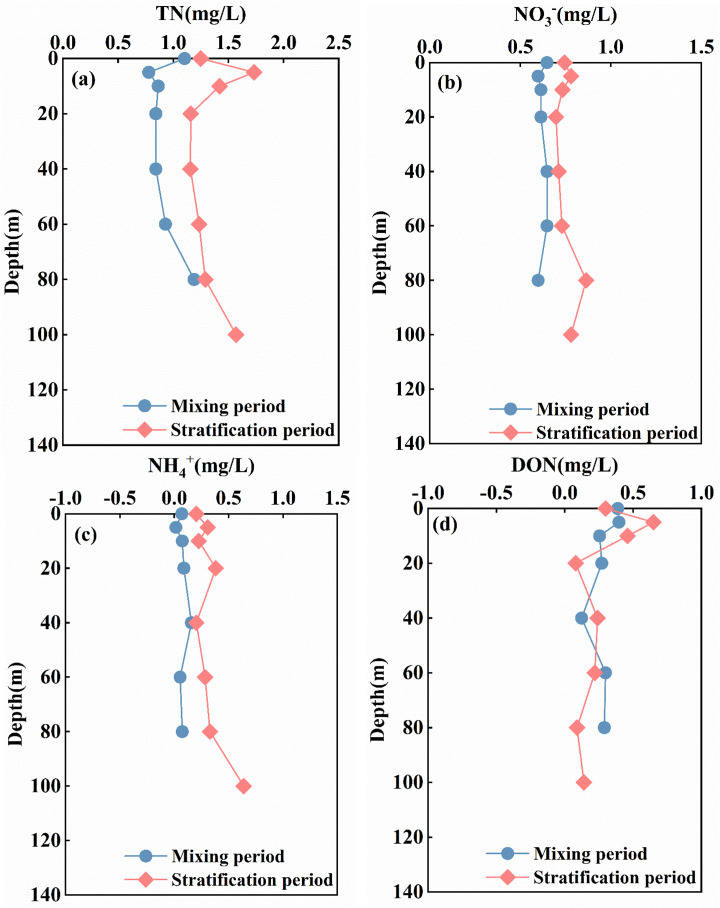
Vertical distribution characteristics of nitrogen in the Longyangxia Reservoir.

The average concentration of total nitrogen (TN) in the reservoir in April was 1.3 ± 0.14 mg/L, which was higher than the 1.0 ± 0.08 mg/L recorded in January. The TN concentration in the reservoir initially decreases and then increases along the length of the reservoir. The concentrations in the upstream and downstream sections are higher than those in the reservoir section (Fig 4). This pattern can be attributed to the slowing of water flow in the reservoir area, which allows more time for various forms of nitrogen to mix with the water, thereby reducing its overall concentration. This observation highlights the impact of dam and reservoir construction on water quality [37]. The difference in vertical total nitrogen (TN) concentration during the mixing period was minimal, with an average value of 0.99 ± 0.06 mg/L. During the stratification period, the TN concentration exhibited significant vertical variation, initially increasing and then decreasing within the 0–20 m range, reaching a maximum of 1.733 mg/L at 5 m. Below 20 m, TN concentration gradually increases with water depth (Fig 5a). The vertical variation range of TN was between 1.234 and 1.733 mg/L, yielding an average of 1.35 ± 0.21 mg/L.

In terms of the occurrence forms of TN, TDN constitutes the predominant form, accounting for over 98% of the total TN, while the concentration of particulate nitrogen (PN) is relatively low. NO_3_- and DON are the primary forms of TDN in water, representing 66.71% and 25.83% of the TDN during the mixing period, and 62.39% and 21.59% during the stratification period, respectively. In general, the concentrations of NO_3_- and DON in the reservoir in April (0.81 mg/l and 0.30 mg/l) were higher than those in January (0.66 mg/l and 0.26 mg/l). Spring marks the onset of the snowmelt period. As temperatures rise, agricultural activities, including planting and animal husbandry around the Longyangxia Reservoir, begin to intensify. This increase in agricultural activity may lead to elevated nitrogen levels in the water. In January, the concentration differences of NO_3_- and DON in water at various depths were minimal, exhibiting no distinct stratification characteristics. In April, the vertical variation trend of NO_3_- and DON content exhibited a pattern similar to that of TN, initially increasing and then decreasing at depths of 0–20 meters, while slightly increasing with greater water depth below 20 meters (Fig 5b and 5d). During the sampling period, the concentration of DO in the Longyangxia water body was elevated. In an aerobic environment, the process of nitrification was pronounced, resulting in the majority of ammonia nitrogen being converted into nitrate nitrogen. In addition, sediment serves as a significant endogenous source of nitrate in aquatic environments. Consequently, deeper water layers may be influenced by nitrification processes and the release of nitrogen from sediments [38]. The concentration of NH_4_+ in LYXR remained low, averaging 0.07 ± 0.05 mg/L in January and 0.21 ± 0.18 mg/L in April. The NH_4_+ content during the stratification period exhibited an increasing trend with depth (Fig 5c). The reduction of the DO level in the bottom water led to the release of ammonia nitrogen from the sediment, with the maximum NH_4_+ concentration (0.64 mg/L) observed at a depth of 100 m below the bottom. The presence of a thermocline and the enhanced assimilation by phytoplankton contributed to the low NH_4_+ concentration in the surface water. NO_2_-, an intermediate product in the nitrogen cycle, is highly susceptible to transformation and is essentially undetectable in reservoir water bodies.

### Distribution characteristics of nitrate nitrogen and oxygen isotopes in the Longyangxia Reservoir

**Fig 6:**
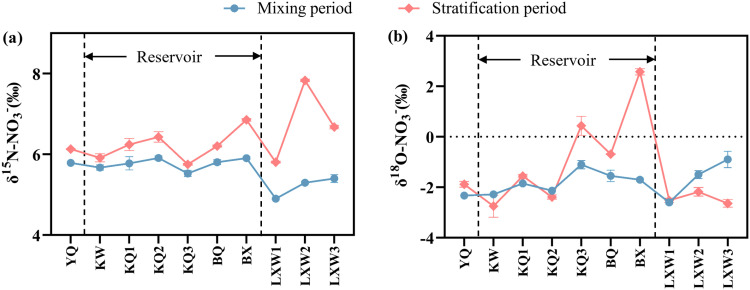
Longitudinal distribution characteristics of δ^15^N-NO_3_^-^ and δ^18^O-NO_3_^-^ in the Longyangxia Reservoir.

**Fig 7:**
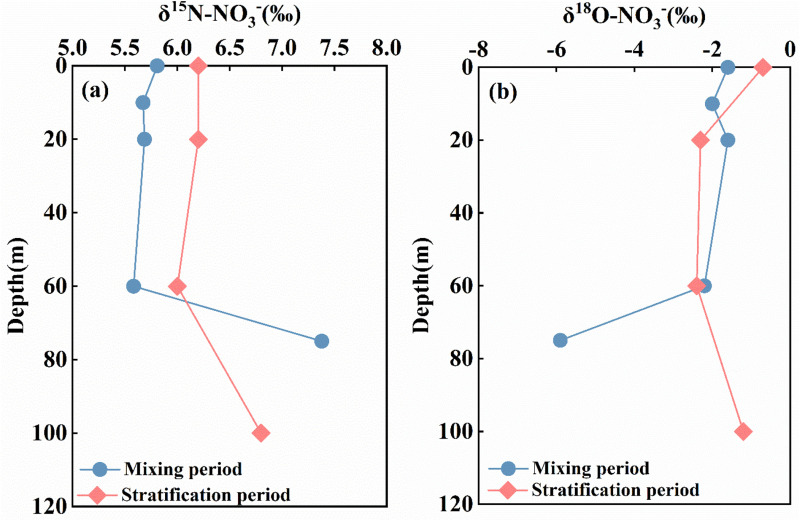
Vertical distribution characteristics of δ^15^N-NO_3_^-^ and δ^18^O-NO_3_^-^ in the Longyangxia Reservoir.

The δ^15^N-NO_3_^-^ value in the reservoir section exhibited relative stability in January, ranging from 5.67‰ to 5.91‰, with an average value of 5.89‰. The average δ^15^N-NO_3_^-^ value in the downstream section was 5.4‰. The δ^15^N-NO_3_^-^ values in the reservoir section ranged from 5.8‰ to 6.8‰ in April, with an average value of 6.22‰. The average δ^15^N-NO_3_^-^ value in the downstream section was 6.79‰. The average δ^15^N-NO_3_^-^ value in April was significantly higher than that in January (Fig 6a). In January, the δ^15^N-NO_3_^-^ values in the 0-60m water column exhibited minimal variation, while below 60m, the δ^15^N-NO_3_^-^ values increased significantly with depth. In April, the δ^15^N-NO_3_^-^ value of surface water (0–20 m) remained relatively stable at 6.2 ‰, which was higher than the value recorded in January. The δ^15^N-NO_3_^-^ value of the middle water body (20–40 m) exhibited a decreasing trend with increasing depth, while the δ^15^N-NO_3_^-^ value of the bottom water body (40–100 m) showed an increasing trend with depth (Fig 7a).

The δ^18^O-NO_3_^-^ in the reservoir section varied from −2.29‰ to −1.1‰ in January, with a mean value of −1.79‰, and the mean value of δ^18^O-NO_3_^-^ in the downstream section was −1.7‰, indicating an increasing trend along the flow direction. The δ^18^O-NO_3_^-^ values varied between −2.8‰ and 0.4‰ in the reservoir section in April, with a mean value of −1.39‰. The δ^18^O-NO_3_^-^ values in the downstream section ranged from −2.6‰ to 2.6‰, with a mean value of −1.19‰. The δ^18^O-NO_3_^-^ values were higher in April than in January (Fig 6b). In January, the δ^18^O-NO_3_^-^ values in the 0–20 meters water column exhibited a trend of initially decreasing followed by an increase. Below 20 m, the δ^18^O-NO_3_^-^ values significantly decreased with increasing depth. In April, the δ^18^O-NO_3_^-^ values in the surface water were higher than those at other depths. The δ^18^O-NO_3_^-^ values in the middle water column decreased with increasing depth, while that in the bottom water column increased with depth (Fig 7b).

## Discussion

### Effects of environmental factors on various forms of nitrogen in Longyangxia Reservoir

**Fig 8:**
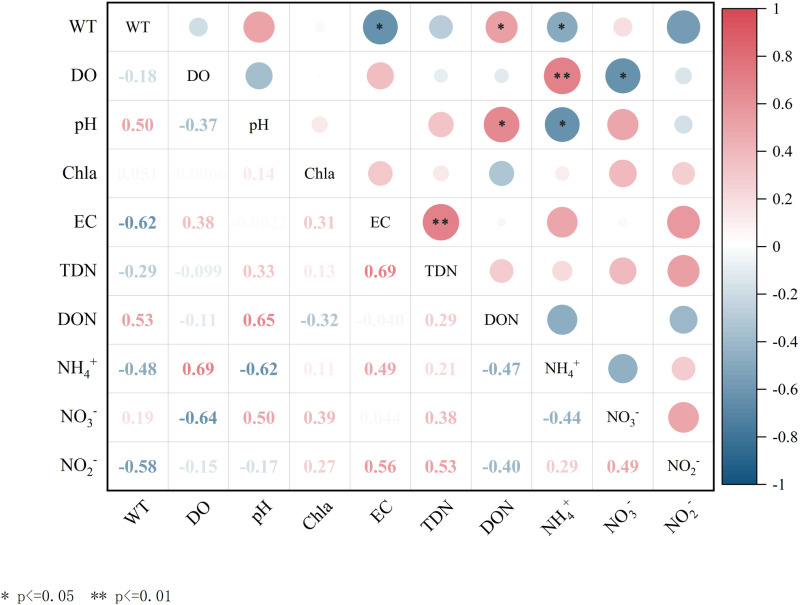
Correlations between nitrogen and physical and chemical indicators in the Longyangxia Reservoir during the stratification period.

**Fig 9:**
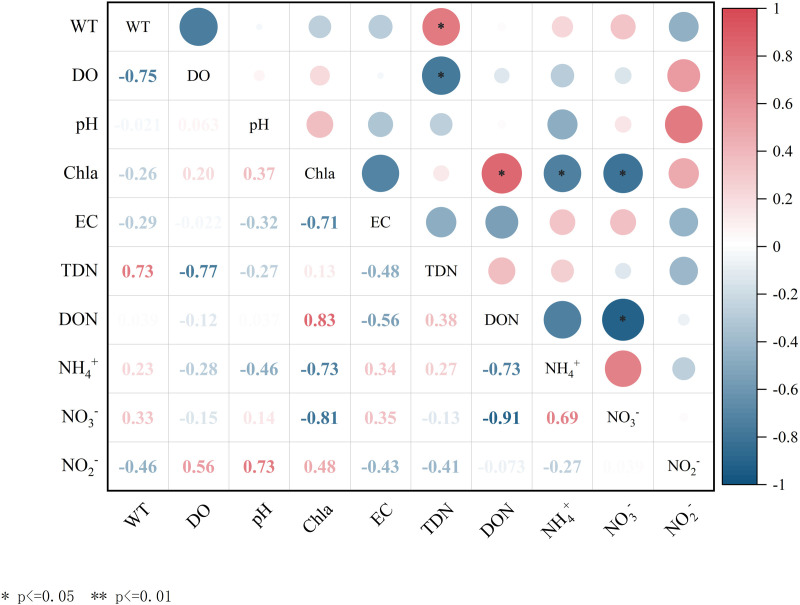
Correlations between nitrogen and physical and chemical indicators in the Longyangxia Reservoir during the mixing period.

The distribution and transformation of nitrogen forms in water bodies are influenced not only by the input of exogenous nitrogen and the release of endogenous nitrogen in lakes but also by a series of water environmental factors, including water temperature and dissolved oxygen content [13,17]. The primary sources and distribution characteristics of various forms of nitrogen in the study area exhibit significant temporal variation. Therefore, it is essential to investigate the correlation between environmental factors, such as WT, DO, pH, and the various forms of nitrogen. This study aims to analyze the correlation between various forms of nitrogen and environmental factors in the LYXR. The objective is to elucidate the environmental factors influencing the transformation of nitrogen forms during both stratification and mixing periods. The results of the correlation analysis concerning different nitrogen forms and environmental factors in the LYXR water during these periods are presented in Figs 8 and 9.

WT was positively correlated with DON (p < 0.05) and negatively correlated with NH_4_+ (p < 0.05) in the LYXR during the stratification period. Water temperature influences biological and chemical reactions in the water column and usually causes changes in other water quality indicators. During the stratification period, photosynthesis was vigorous, and the increase in DO concentration and WT fostered conditions conducive to the growth and reproduction of plankton and aquatic plants [39]. Green plants and plankton absorb and utilize NH_4_+ and NO_3_-, which reduces the concentration of NH_4_+ while increasing the concentration of Chl-a in the water. The correlations between WT, various forms of nitrogen and Chl-a also indicated that nitrogen assimilation was significantly enhanced by the involvement of aquatic organisms during the stratification period. DO was significantly positively correlated with NH_4_+ (p < 0.01) and negatively correlated with NO_3_- (p < 0.05). Dissolved oxygen is a critical factor in determining whether water exists in an anaerobic environment, significantly influencing nitrification and denitrification processes. During the stratification period, the average WT of the reservoir was 8.16 °C, with an average DO concentration of 7.85 mg/L, conditions that are favorable for nitrification. The nitrification process consumes dissolved oxygen. Thus, an increase in NO_3_- concentration and a decrease in NH_4_+ concentration will lead to a reduction in DO levels in the water. During the stratification period, the algal population in the water experienced rapid growth, leading to an increase in DON content. Concurrently, algae absorb CO_2_ and release oxygen during photosynthesis, which enhances the pH level of the water. Consequently, a significant positive correlation was observed between pH and DON (p < 0.05), while a significant negative correlation was found between pH and NH_4_+ (p < 0.05). Electrical conductivity (EC) exhibited a significant positive correlation with TDN (p < 0.01). EC is closely associated with the concentration of nutrients in water. An increase in EC indicates an increase in the NO_3_- concentration in water [40].

There was a positive correlation (p < 0.05) between WT and TDN in the LYXR during the mixing period. Temperature primarily influences the mutual transformation and content changes of various nitrogen forms in water by affecting the metabolic activities of microorganisms [25]. During winter, the average temperature of aquatic environments decreases, resulting in reduced microbial activity. This decline in microbial activity inhibits the nitrogen cycling processes, ultimately leading to a reduction in the concentrations of various forms of nitrogen in the water.DO was negatively correlated with TDN (p < 0.05). The DO content in the LYXR was relatively high during the mixing period, with an average concentration of 8.78 mg/L, which facilitates nitrification. The nitrification process consumes DO. Therefore, an increase in TDN concentration in water results in a decrease in DO concentration. In winter, as water temperatures decrease, the metabolic activities of aquatic plants and algae diminish, which may ultimately result in their death. Consequently, their uptake of nitrogen nutrients from the water declines. Simultaneously, organic nitrogen within organisms is decomposed into inorganic nitrogen, which is subsequently released into the water, leading to an increase in NH_4_+ and NO_3_- [41]. Thus, Chl-a was negatively correlated with NH_4_+ and NO_3_- (p < 0.05) and positively correlated with DON (p < 0.05).

Additionally, the influence of physical factors such as solar radiation intensity and the location of water inlets and outlets on various forms of nitrogen in the water body of large and deep reservoirs has also attracted widespread attention [30]. On the one hand, solar radiation determines the magnitude of water temperature. In April, with the increase of solar radiation, the temperature difference between the surface and the bottom of the water gradually increases, and the thermal stratification gradually forms. This means that the influence of temperature on thermal stratification is mainly controlled by solar radiation. On the other hand, solar radiation also promotes the growth of algae, which accelerates the absorption of dissolved inorganic nitrogen (NO_3_- and NH_4_+) and then converts it into DON. Therefore, solar radiation will alter the concentrations of various forms of nitrogen by affecting water temperature stratification, algal activity, and microbial processes. The regulation mode of reservoir storage significantly influences water quality. Research indicates that the bottom release of water preferentially discharges the hypoxic bottom water, which contains accumulated NH_4_+ and N_2_O. This process can lead to the direct introduction of these pollutants into downstream water bodies, thereby increasing the risk of water quality degradation [42]. To mitigate the ecological impact resulting from the low-temperature water discharged from the bottom layer, the LYXR has predominantly employed the middle hole discharge method in recent years.

### Migration and transformation of NO_3_^-^ in the Longyangxia Reservoir

Changes in the nitrogen content and δ15N and δ18O values of dissolved NO_3_- were influenced mainly by the effects of mixing various nitrogen sources and transformations of various forms of nitrogen. The transformation of NO_3_- mainly includes nitrification, denitrification and assimilation [43]. When NO_3_- is assimilated and absorbed by plants, the ratio of δ15N- NO_3_- to δ18O-NO_3_- tends to be 1 as the δ15N value of the remaining NO_3_- increases [44]. The ratios of δ15N-NO_3_- and δ18O-NO_3_- in reservoir waters during the sampling period of this study were generally greater than 1. These findings suggest that phytoplankton-mediated NO_3_- assimilation is not a major process. Plant assimilation follows a pattern of nitrogen assimilation in which NH_4_+ is utilized first, followed by NO_3_-.

**Fig 10:**
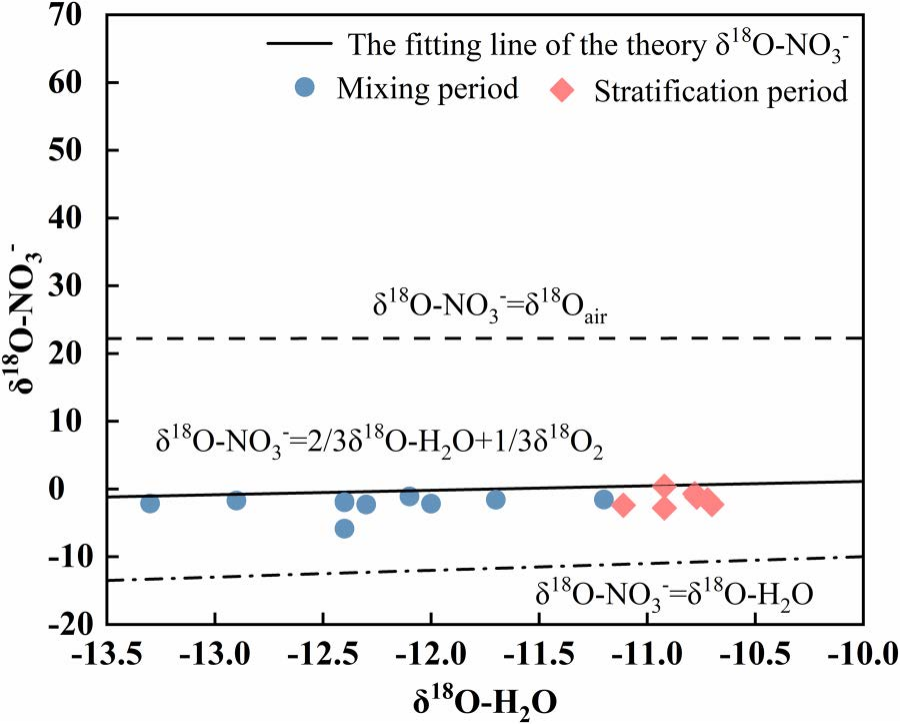
Relationship between δ^18^O-H_2_O and δ^18^O-NO_3_^-^ in water during the sampling period in the study area.

Nitrification refers to the process of the gradual oxidation of ammonia nitrogen to nitrate, which is an important method of nitrogen conversion in water. The δ18O-NO_3_- value of NO_3_- produced by nitrification ranges from −10‰ to 10‰ [45]. In this study, the δ18O-NO_3_- isotope eigenvalue domain of the water samples during the mixing period ranged from −5.9‰ to −1.1‰. The δ18O-NO_3_- values of the water samples during the stratified period ranged from −2.8‰ to 2.6‰. All of these values are within the typical range of nitrification. The δ15N-NO_3_- values ranged from 5.58‰ to 7.8‰, indicating that the NO_3_--N in the Longyangxia Reservoir may have originated mainly from the nitrification of soil organic nitrogen. In addition, microorganisms can oxidize NH_4_+ to NO_3_- during nitrification. In the absence of kinetic fractionation, 2/3 of the oxygen atoms in nitrate produced by nitrification come from the surrounding water column, and 1/3 come from atmospheric O_2_ [45]. In this study, the measured δ18O-H_2_O values of the Longyangxia Reservoir ranged from −13.3‰ to −10.7‰. Combined with the typical value of atmospheric δ18O-O_2_ of 23.5‰ [46]. The δ18O-NO_3_- values of the theoretical nitrification products of the reservoir ranged from −0.78‰ to 0.43‰. The δ18O-NO_3_- values of most of the water samples from Longyangxia were near the theoretical values (Fig 10). These findings further indicate that the nitrification reaction was the main biogeochemical process affecting the nitrogen cycle in the water body of the Longyangxia Reservoir during the sampling period. The observed δ18O-NO_3_- value of the Longyangxia reservoir water was lower than that of the theoretical nitration reaction. The possible reason is that the degree of exchange of O atoms between NO_2_- and H_2_O during the nitrification process of the soil affects the δ18O abundance of the generated NO_3_-, making it lower than the theoretical range [47].

**Fig 11:**
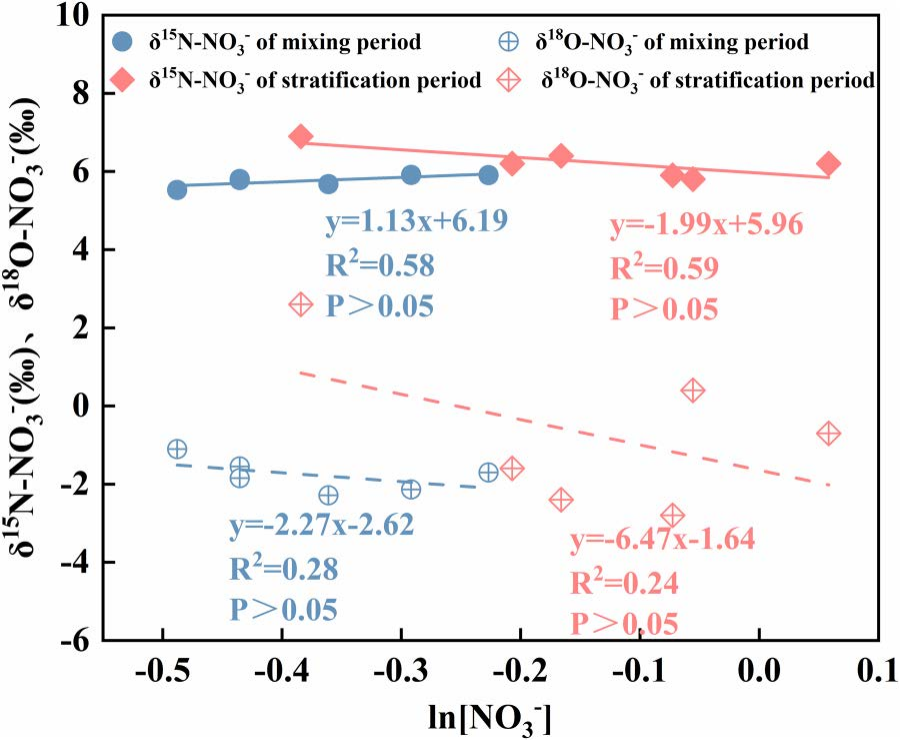
Regression curves of ln(NO_3_^-^) with δ^15^N-NO_3_^-^ and δ^18^O-NO_3_^-^ in the study area.

Denitrification is the process by which microorganisms use organic carbon as an electron donor and nitrate as an electron acceptor to reduce nitrate to N_2_O and N_2_, thereby removing nitrogen from the ecosystem. Denitrification is a key process in maintaining ecosystem health and controlling eutrophication [18]. When denitrification occurs, NO_3_- is consumed, resulting in increases in δ15N-NO_3_- and δ18O-NO_3_- in the remaining NO_3_-. When the δ15N-NO_3_-/δ18O-NO_3_- ratio is between 1.3 and 2.1, denitrification occurs [48]. The δ15N-NO_3_-/δ18O-NO_3_- ratios of the water samples from the Longyangxia Reservoir in this study were not in the range of 1.3 to 2.1. The δ15N-NO_3_- value (6.8‰) at 100 m during the stratification period of the reservoir was greater than the δ15N-NO_3_- value (5.8‰ to 6.2‰) from 0–80 m, and the NO_3_- content decreased. This may be due to the occurrence of denitrification. However, denitrification mainly occurs in anoxic or hypoxic environments with DO levels below 2 mg/L [49]. Although the DO concentration in the bottom water was lower than those in the surface and middle waters (Fig 3b), it still did not support the occurrence of denitrification. This indicates that there are other reactions in the bottom water that affect the NO_3_- content and its isotope value. The bottom water is in close proximity to the sediments, which contain large amounts of nitrogen and have high levels of δ15N-NO_3_-, which are easily released into the overlying water. In addition, the classical Rayleigh equation is capable of describing changes in the isotopic composition of the remaining reactants during isotopic fractionation. Therefore, the presence of denitrification in the water bodies of this region can be determined based on the negative correlation of δ15N-NO_3_- and δ18O-NO_3_- values with ln(NO_3_-) [50]. There was no statistically significant negative correlation (p < 0.05) between reservoir water NO_3_- concentrations and their δ15N-NO_3_- and δ18O-NO_3_- values during the sampling periods (Fig 11). These results demonstrated that denitrification was weak in the LYXR.

### Main sources of NO_3_^-^ in the Longyangxia Reservoir and the contribution of each source

**Fig 12:**
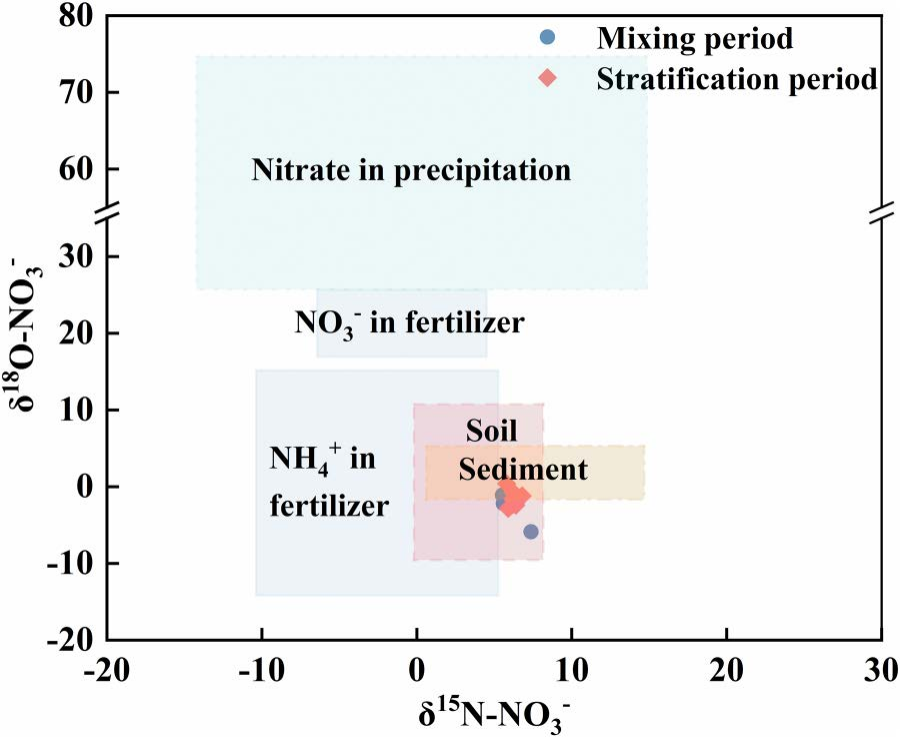
δ^15^N-NO_3_^-^ and δ^18^O-NO_3_^-^ ranges of different sources.

Based on the surroundings of the study area, the potential sources of nitrate in the water of the Longyangxia Reservoir were atmospheric precipitation, soil organic nitrogen, chemical fertilizers and sediment. Because the nitrogen and oxygen isotope eigenvalues of nitrate differ between different nitrogen sources, the characteristic ranges of nitrogen and oxygen isotopes can be used to qualitatively identify the source of nitrate in the water [18]. The nitrogen and oxygen isotope ranges for nitrate from different typical sources were obtained from previous studies [45]. The nitrogen and oxygen isotope values of nitrate in the water samples from the Longyangxia Reservoir were partly located in the soil organic nitrogen region and partly in the ammonium nitrogen fertilizer region, whereas those of the other samples fell in the sediment region (Fig 12). These findings indicate that nitrate in the Longyangxia Reservoir is the result of the mixing of multiple sources, in which soil organic nitrogen is the main source. According to previous studies, nitrate nitrogen in the Yellow River system is derived mainly from soil organic nitrogen mineralization, chemical fertilizers and sewage/manure [51]. The results of this study are consistent with the above findings.

**Fig 13:**
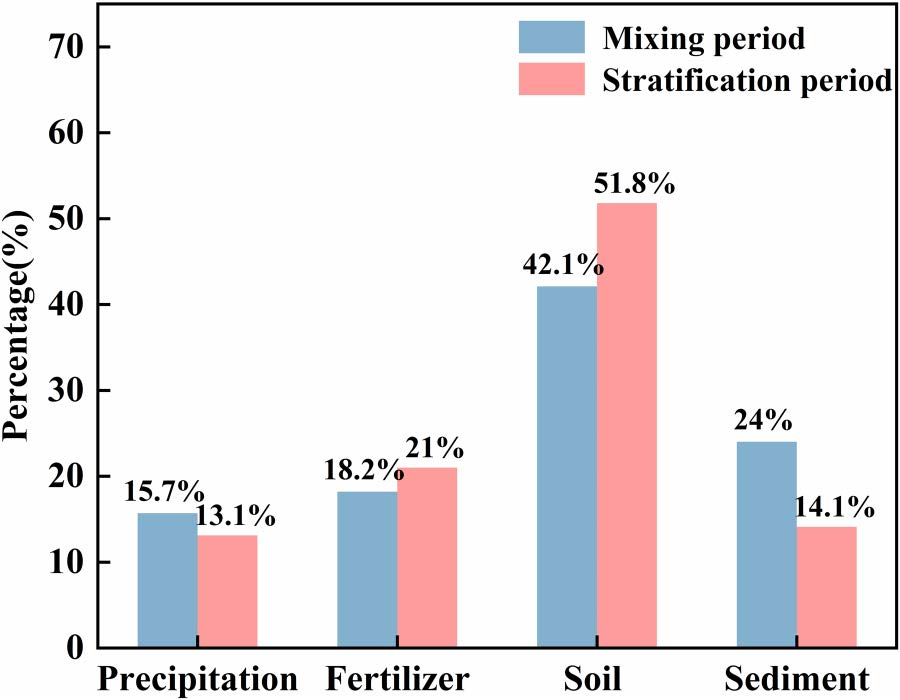
Contribution rates of each nitrate source during the sampling period in the Longyangxia Reservoir.

The nitrogen and oxygen isotopes of nitrate combined with the SIAR model can be used to quantify the contribution of each source of nitrate in the water body of the Longyangxia Reservoir. It has been shown that mineralization and nitrogen fixation occurring during nitrogen transport and transformation are less isotropic. When the pH ≥ 9.3, NH_4_+ is converted to NH_3_, and ammonia volatilization needs to be considered [19]. The pH values of most point waters in this study were less than 9.3; therefore, isotopic fractionation due to ammonia volatilization was not considered. In addition, the NH_4_+$C_{jk}$ in the SIAR model was set to zero [45]. The output of the SIAR model revealed that the overall contributions of the four types of nitrate sources in the Longyangxia Reservoir were in the following order during the mixing period: soil organic nitrogen (42.1%)> sediment (24%)> fertilizer (18.2%)> atmospheric precipitation (15.7%) (Fig 13). The overall contribution of nitrate sources during the stratified period was in the following order: soil organic nitrogen (51.8%)> fertilizer (21%)> sediment (14.2%)> atmospheric precipitation (13.1%) (Fig 13).

According to relevant research, the Gonghe Basin, located to the west of the LYXR, contributes a significant amount of sediment to the reservoir annually. Within the watershed controlled by the reservoir dam, the average annual sediment transport over the years is approximately 23.08 million tons [52]. Furthermore, the rise in water level due to the impoundment of the Longyangxia Reservoir has increased the frequency of contact between the river and the riparian soil. Consequently, soil organic nitrogen has become the primary source of contribution. Agricultural land constitutes one of the primary land-use types in the study area, necessitating fertilization to satisfy the growth demands of crops and to enhance soil fertility. In the northern region, the agricultural irrigation period occurs from April to May, during which chemical fertilizers are introduced into the soil alongside irrigation water. These fertilizers subsequently enter the reservoir water body through river flow and stormwater runoff. Notably, the frequency of precipitation events during the stratification period is typically higher than that observed during the mixing period. Consequently, the contribution of chemical fertilizers to reservoir NO_3_- levels is greater during the stratification period (21%) compared to the mixing period (18.2%). Previous studies have generally posited that atmospheric deposition contributes minimally to the NO_3_- levels in rivers and lakes [53]. First, NO_3_- represents a small proportion of the dissolved inorganic nitrogen found in atmospheric deposition, with NH_4_+ constituting a comparable or even greater proportion. Second, the NO_3_- that is directly deposited from the atmosphere into water bodies accounts for only a minor fraction of the total nitrate present in these water bodies. NO_3_- from the atmosphere may percolate into the soil and accumulate in groundwater, or it may be intercepted and absorbed by plants and microorganisms within the catchment area. Atmospheric precipitation contributed the least to the concentration of NO_3_- in the water in this study. This is attributed to the fact that the NO_3_- deposited directly from the atmosphere constitutes only a minor fraction of the total NO_3_- present in the water. The distribution of nitrate in aquatic environments is primarily influenced by indirect processes [54]. The research conducted by Jin on the contribution rate of nitrate sources in the water of the typical deepwater reservoir, Qiandao Lake, indicates that sediment, as a significant endogenous source of nitrate, contributes 12.3% and 15.1% to the water body, respectively [40]. In a parallel study, Wang analyzed the sources of nitrate in the water body of Hongfeng Reservoir, situated in a subtropical karst area, and found that the degradation of organic nitrogen from sediment accounted for a higher proportion of nitrate in the lake system, with contribution rates of 38% in April and 24% in August [14]. Thus, the influence of sediment nitrogen release on nitrate levels in the water bodies of large and deep reservoirs is significant and should not be overlooked. In this study, the contribution rate of sediment to nitrate in water was found to be 14.1% during the stratification period and 24% during the mixing period. The contribution from endogenous sources during the mixing period was greater than that during the stratification period, as the dissolved oxygen (DO) levels were higher in the mixing period than in the stratification period, leading to a greater release of nitrate from the water-sediment interface under aerobic conditions [14].

**Fig 14:**
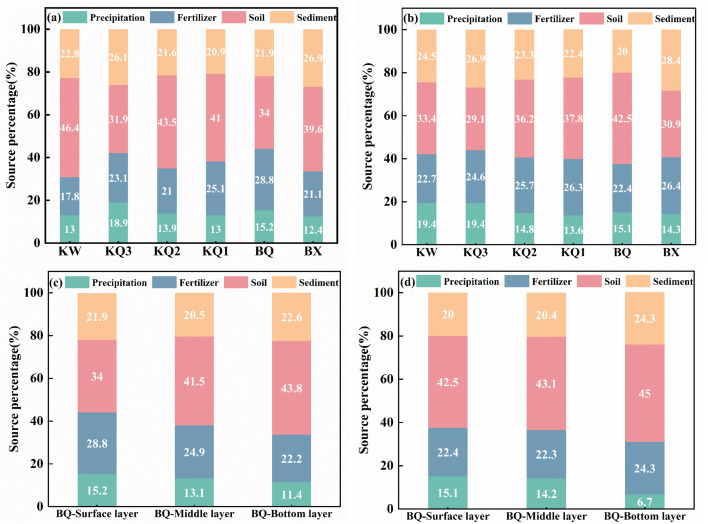
Contributions of the four types of nitrate sources in the Longyangxia Reservoir at each sampling point ((a) and (c) contributions from the sampling points along the reservoir and in the vertical direction in front of the dam during the mixing period; (b) and (d) contributions from the sampling points along the reservoir and in the vertical direction in front of the dam during the stratification period).

In terms of the contribution of nitrate sources from each sampling point (Fig 14), the contributions of soil organic nitrogen in front of the reservoir dam and in the reservoir area were greater than those at the other points. These results are attributed to reservoir impoundment due to artificial regulation. The rise of the reservoir water level increases the frequency of contact between the water body and the soil in the riparian zone and the scouring process [21]. The average contribution rates of soil organic nitrogen in the stratified and mixing periods were 42.5% and 34%, respectively. The average contribution rates of soil organic nitrogen in the reservoir middle water were 43.1% and 41.5%, respectively. The average contribution rates of soil organic nitrogen in the reservoir bottom water were 45% and 43.8%, respectively. The contribution rate of soil organic nitrogen in the bottom layer of the reservoir was greater than those in the surface and middle layers. The probable reason for this is that the damming slowed the flow, allowing these organics to settle and be stored in the bottom sediment, resulting in an increased bottom contribution. Owing to the great depth of the Longyangxia Reservoir, nitrogen release from the sediment has a greater impact on the bottom water and a lesser impact on the middle and upper water layers. Its contribution to nitrate is greater in bottom waters than in surface and middle waters. In addition, owing to the long hydraulic residence time caused by dam construction, the contribution rates of other nitrogen sources at each point in the reservoir exhibit little seasonal difference.

In summary, while the current level of water pollution in the Longyangxia basin is relatively low, there are still potential pollution risks in the water environment that could threaten future water quality. Generally, the remediation costs associated with polluted areas are substantial, making the prevention of water pollution a more effective strategy than remediation. Therefore, by analyzing the temporal and spatial distribution differences and sources of nitrate in the LYXR, it is recommended to enhance regional vegetation coverage. This approach aims to prevent or mitigate hydraulic and wind erosion of exposed surface soil, thereby reducing the contribution of soil organic matter to NO_3_^-^ levels in water during rainfall events or reservoir impoundment. Endogenous pollution resulting from sediments warrants significant attention. The nitrogen present in these sediments may originate from the rainbow trout cage culture area in the LYXR. During the cage culture of rainbow trout, 72% to 79% of the nitrogen in the feed is released into the water body and subsequently deposited in the sediments, primarily due to low utilization rates and the feces excreted by the fish. Additionally, various cage disinfectants and fish vaccines present a pollution threat to the aquatic environment. Therefore, it is necessary to reasonably plan the cage culture area and regulate the scale of breeding. Another key measure is optimizing agricultural fertilization. By promoting precision fertilization technology, reducing the use of chemical fertilizers, and encouraging farmers to utilize organic fertilizers instead, nitrogen leaching can be effectively controlled, thereby reducing the contribution of agricultural fertilizers to nitrate pollution.

## Conclusions

This study examines the dynamics and sources of nitrate under the thermal stratification conditions of typical deep reservoirs in the upper reaches of the Yellow River. The LYXR exemplifies a typical warm-monomictic lake. In January, the water body exhibited a vertical mixing state, while thermal stratification began to develop in April. The thermal stratification of water significantly influences the vertical distribution characteristics of DO, Chl-a, and various forms of nitrogen in the LYXR. Notably, the Chl-a content in the deeper layers exhibited a phenomenon known as the deep chlorophyll maximum (DCM), which occurred at a pronounced water temperature gradient. The DO levels in the water body were influenced by phytoplankton photosynthesis, resulting in a slight increase within the thermocline. Furthermore, the thermocline impedes vertical mixing of the water, leading to a nitrate concentration profile characterized by ‘upper low, middle high, and lower high.’ The primary forms of nitrogen in the LYXR were identified as NO_3_^-^ and DON during various periods. Throughout the sampling period, the range of δ^15^N-NO_3_^-^ varied from 5.58 ‰ to 7.38 ‰, while the range of δ^18^O-NO_3_^-^ fluctuated between −5.87 ‰ and −2.58 ‰. The concentration of DO exceeded 2 mg/L. In general, nitrification is the most significant transformation process of nitrogen in LYXR. Denitrification is absent in bottom waters due to aerobic conditions, and the dynamics of nitrate are influenced by both nitrification and the release of nitrogen from sediments. In addition, soil organic nitrogen serves as the primary source of nitrate in the reservoir, with contribution rates of 51.8% during the stratification period and 42.1% during the mixing period. Influenced by agricultural activities, the contribution rate of chemical fertilizer during the stratification period is 21%, making it the second highest source after soil nitrogen. Furthermore, due to enhanced hydrodynamic conditions and changes in the aerobic environment during the mixing period, the contribution rate of sediment is also significant at 24%.

This study also has certain limitations. The data collected is relatively small, which may restrict the phenomena and conclusions that can be drawn from it. In future studies, increasing the sampling frequency will allow for a more comprehensive understanding of the seasonal variations affecting the geochemical processes and source apportionment of nitrate in the study area. In addition, isotope fractionation is a critical consideration in the identification of nitrogen sources. The fractionation factor was set to zero due to the absence of significant denitrification in the studied water; however, this assumption deviated from the actual conditions in the study area. Therefore, it is essential to determine the isotope fractionation factors for various end members in future work to minimize the uncertainty in nitrate tracing results.

## Supporting information

S1 Table Geographical coordinates of sampling points. (XLSX)

S2 Table Physicochemical parameters and isotopic characteristics of water at sampling points in Longyangxia Reservoir during mixing period (Mean ± Standard Deviation). (XLSX)

S3 Table Physicochemical parameters and isotopic characteristics of water at sampling points in Longyangxia Reservoir during stratification period (Mean ± Standard Deviation). (XLSX)

S4 Table Contribution of potential nitrate sources at sampling points in Longyangxia Reservoir during mixing period. (XLSX)

S5 Table Contribution of potential nitrate sources at sampling points in Longyangxia Reservoir during stratification period. (XLSX)
